# Unilateral Maxillary Canine Agenesis: A Case Report and Literature Review

**DOI:** 10.1155/2014/685014

**Published:** 2014-08-11

**Authors:** Nagihan Koç, L. Berna Çağırankaya, Nursel Akkaya

**Affiliations:** Department of Dentomaxillofacial Radiology, Faculty of Dentistry, Hacettepe University, Sihhiye, 06100 Ankara, Turkey

## Abstract

Congenital absence of maxillary permanent canines is an extremely rare condition, which may appear as part of a syndrome or as a nonsyndromic form. Nonsyndromic canine agenesis combined with other types of tooth agenesis has occasionally been described in the literature but isolated cases are rarely observed. This report presents an isolated case of maxillary permanent canine agenesis in a healthy 18-year-old female patient and a literature review on the prevalence, etiology, and differential diagnosis of the condition.

## 1. Introduction

Tooth agenesis or hypodontia is one of the most common anomalies of the human dentition, which is characterized by the developmental absence of one or more teeth. The condition can occur in association with a recognized genetic syndrome or as a solitary anomaly (nonsyndromic). Lack of one or a few permanent teeth without any systemic disorder is the most common phenotype of hypodontia [1].

Both environmental and genetic factors can cause the failure of tooth development although in the majority of the cases hypodontia has a genetic basis [1].

Recent reports have shown that, in the Caucasian population, the prevalence of hypodontia in permanent dentition (third molar excluded) is about 4.5–7.4% [2].

In syndromic oligodontia, the permanent canines are often reported as missing though being with a low frequency [3]. Congenital absence of permanent canines was occasionally reported in cases of nonsyndromic patients with advanced hypodontia or oligodontia [4–7] but isolated cases of maxillary permanent canine agenesis are rare [8–10]. Previous studies showed that the prevalence of the maxillary permanent canine agenesis varies between 0.07 and 0.13% [11].

This case report presents a unilateral maxillary permanent canine agenesis. A literature review on the prevalence, etiology, and diagnosis of the condition was also carried out.

## 2. Case Report

**Figure 1:**
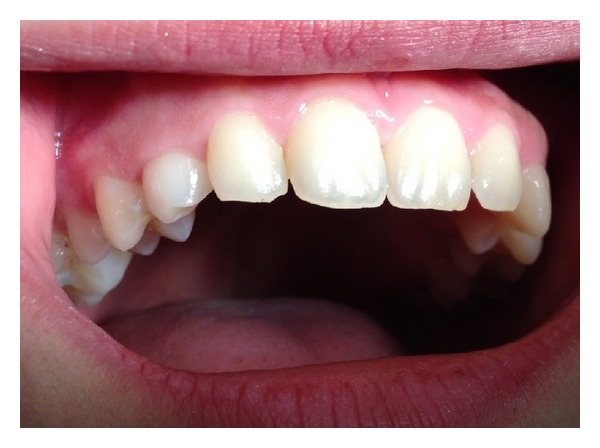
Intraoral view of the maxillary arch showing the small canine on the right side with signs of attrition.

An 18-year-old female patient presented to the Department of Dentomaxillofacial Radiology, Hacettepe University, for a routine dental examination in October 2013. She was in good health with no history of systemic disease or syndrome. Intraoral examination revealed a class I molar relationship with a centered dental midline and increased anterior overbite. A diastema of 2 mm between 22 and 23 was observed. Clinical examination of the maxillary teeth revealed a small canine showing signs of attrition on the right side with no mobility (Figure 1). There was a small caries on the distal aspect of the tooth. In order to detect permanent canine, palpation of the labial sulcus and the palatal side of the alveolar process failed to indicate the presence of unerupted canine.

**Figure 2:**
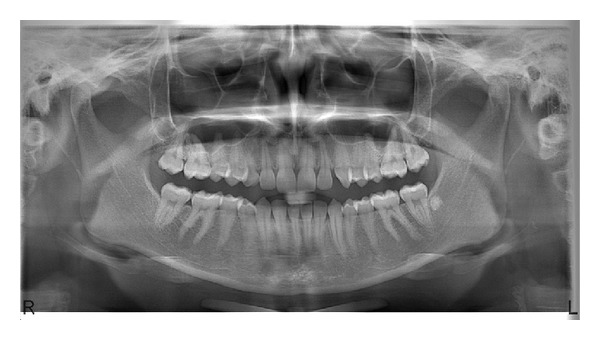
Panoramic radiograph showing the absences of right permanent maxillary canine and all third molars. The deciduous canine is persisting with external root resorption. Note the radiopaque lesion in the left mandibular third molar region.

**Figure 3:**
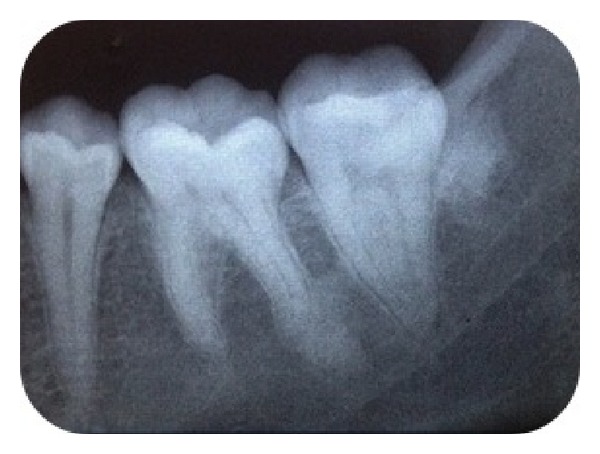
Periapical radiograph of the left mandibular molar region, showing the osteosclerotic area.

A panoramic radiograph was taken to detect the presence and the location of the permanent canine and to check for any other anomalies. The radiograph showed that the right permanent canine was missing and the erupted canine was deciduous with its small root, short crown size, and thin enamel structure. The deciduous canine was persisting with root resorption grade 1 [12] and all third molar germs were also missing (Figure 2). The clinical history that ruled out any possibility of the permanent canine had been extracted or the patient had undergone dental trauma. There was no relevant family history for this condition. No other tooth anomalies were observed. However an irregularly shaped radiopacity in the left third molar region of the mandible was remarkable (Figure 2). The lesion was diagnosed as idiopathic osteosclerosis on periapical view (Figure 3).

The patient was referred to the Department of Restorative Dentistry for the restoration of the decayed tooth.

## 3. Discussion

Many studies have researched developmental disorders and explained these by using anatomic and evolutionary models. According to Bolk's [13] theory of terminal reduction, due to the phylogenetic evolution of mankind, the reduction of the distal element of a tooth group occurs more frequently than mesially placed teeth: so the most common missing teeth are the second premolars, the upper second incisors, and the third molars. In adapting Butler's field concept to the human dentition, Dahlberg defined the canine as a key tooth that stands alone in its own field displaying great stability and therefore is rarely congenitally missing [14].

The cause of congenital absence of the teeth is variable. Severe hypodontia is usually associated with genetic disorders such as Witkop syndrome, ectodermal dysplasia, and Rieger syndrome [15]. Mild to moderate hypodontia may occur due to early irradiation of tooth germs, various kinds of trauma of the dental region, Down syndrome, and syndromes associated with cleft lip or palate [1].

The etiology of tooth agenesis has generated much debate. Graber [16] claims that congenital absence of teeth is a heritable phenomenon and probably most often passed to each generation by an autosomal dominant pattern with incomplete penetrance and variable expressivity. Brook [17] suggested a multifactorial etiology of hypodontia combining polygenic and environmental influences. The role of genetics has been confirmed but there is a controversy on the influence of environmental factors in monozygotic twin studies: Markovic [18] studied the pattern of the hypodontia in twins and found one member of a monozygotic pair showing unilateral canine agenesis with a mirror-image similarity, which may indicate the genetic bases of hypodontia. However, Kindelan et al. [19] demonstrated that genetic coding is not the sole controlling factor in tooth agenesis in their study of monozygotic twins representing differences in facial appearance and extent of hypodontia. Recently, isolated hypodontia of the maxillary permanent canines is suggested to be associated with mutations in WNT10A gene [20].

The developmental absence of permanent canines is reported to be higher in women and mostly maxilla affected with the left side [8, 10, 21]. Statistical data on maxillary canine agenesis differs in the literature. Harris and Clark [22] found only two cases of congenitally missing maxillary canines among 600 Black American people (0.4%), whereas they could not detect any cases among 1100 White people in the same study. Cho et al. [8] described developmentally absent upper permanent canines in 32 cases among 69.852 Chinese children. Fukuta et al. [21] found only 42 cases with the prevalence of 0.13% in the files of 35.927 outpatients. Davis [23] also reported five such cases out of 1093 Chinese children in her study (0.46%). In European studies, Fekonja [24] recorded one case of maxillary canine agenesis in 212 patients who had undergone orthodontic treatment (2.1%), whereas Rózsa et al. [10] found the prevalence of permanent maxillary canine agenesis 0.27% in their study. Sisman et al. [25] demonstrated that the prevalence was 0.37% in their study on Turkish orthodontic patients.

It was previously reported that single canine agenesis is more predominant than multiple canine agenesis and mostly occurs with other types of dental anomalies such as congenital absence of other teeth, microdontia, delayed tooth formation and eruption of permanent teeth, supernumerary teeth, odontoma, taurodontism, and talon cusps [21]. In our case there was neither congenital absence of other permanent teeth nor other dental anomalies associated with canine agenesis.

There are other possibilities that should be considered in such cases when permanent canines are clinically found to be absent. If the permanent canine could not be palpated in the buccal sulcus by eleven years of age, ectopic eruption and impaction of the teeth must be considered [26]. Bone diseases, cysts, and tumors can cause ectopic eruption and impaction of the maxillary canines. Transposition may occur with the first premolars or lateral incisors although it is a rare finding [27]. Migration of the maxillary canine across the midline which is known as transmigration is an infrequent disorder which might be considered when a permanent canine is clinically missing [28]. Early detection of impacted or missing maxillary canines may enable interceptive treatment and reduce the treatment time, complexity, and complications. Therefore when a permanent canine is clinically found to be missing, a radiographic investigation is essential to determine the presence and localization of the tooth and any associated pathology.

Congenitally missing maxillary canines require a specific treatment plan. Many factors should be considered: the condition of the deciduous teeth, the patient's occlusion (crowding versus spacing and midline deviations of the arch), facial growth pattern, and the preferences of the patient. Treatment options may include the extraction of primary teeth to facilitate spontaneous or orthodontic space closure or retaining the deciduous teeth until the end of growth in order to preserve the alveolar bone quality to provide maximum potential for implant replacement without the need of bone grafting.

In the present case the main aim was to keep the primary canine as far as possible. It was decided to follow up the patient; as the root resorption of the deciduous canine had already begun, referral to a prosthodontist will be needed in the near future.

## References

[1] Arte S (2001). *Phenotypic and genotypic features of familial hypodontia [Ph.D. thesis]*.

[2] Bäckman B, Wahlin YB (2001). Variations in number and morphology of permanent teeth in 7-year-old Swedish children. *International Journal of Paediatric Dentistry*.

[3] Lombardo C, Barbato E, Leonardi R (2007). Bilateral maxillary canines agenesis: a case report and a literature review. *European Journal of Paediatric Dentistry*.

[4] Schalk-van der Weide Y, Bosman F (1996). Tooth size in relatives of individuals with oligodontia. *Archives of Oral Biology*.

[5] Akkaya N, Kiremitçi A, Kansu O (2008). Treatment of a patient with oligodontia: a case report. *Journal of Contemporary Dental Practice*.

[6] Uzuner D, Celik MM, Toy E, Turkdonmez CO (2013). Assessment of hypodontia in the Turkish patients referring to the orthodontic clinic: a retrospective study. *European Journal of Dentistry*.

[7] Moses J, Gurunathan D, Rangeeth BN, Kannan KS (2013). Non-syndromic oligodontia of primary and permanent dentition: 5 year follow up-a rare case report. *Journal of Clinical and Diagnostic Research*.

[8] Cho SY, Lee CK, Chan JCY (2004). Congenitally missing maxillary permanent canines: report of 32 cases from an ethnic Chinese population. *International Journal of Paediatric Dentistry*.

[9] Leong P, Calache H (1999). Bilateral congenitally missing maxillary canines. A case report. *Australian Dental Journal*.

[10] Rózsa N, Nagy K, Vajó Z (2009). Prevalence and distribution of permanent canine agenesis in dental paediatric and orthodontic patients in Hungary. *European Journal of Orthodontics*.

[11] Polder BJ, Van't Hof MA, Van Der Linden FPGM, Kuijpers-Jagtman AM (2004). A meta-analysis of the prevalence of dental agenesis of permanent teeth. *Community Dentistry and Oral Epidemiology*.

[12] Haselden K, Hobkirk JA, Goodman JR, Jones SP, Hemmings KW (2001). Root resorption in retained deciduous canine and molar teeth without permanent successors in patients with severe hypodontia. *International Journal of Paediatric Dentistry*.

[13] Schuurs A (2013). *Developmental Anomalies. Pathology of the Hard Dental Tissues*.

[14] Scott GR, Katzenberg MA, Saunders SR (2008). Dental morphology. *Biological Anthropology of the Human Skeleton*.

[15] Gorlin RJ, Cohen MM, Hennekam RCM (2001). *Syndromes of the Head and Neck*.

[16] Graber LW (1978). Congenital absence of teeth: a review with emphasis on inheritance patterns.. *The Journal of the American Dental Association*.

[17] Brook AH (1984). A unifying aetiological explanation for anomalies of human tooth number and size. *Archives of Oral Biology*.

[18] Markovic M (1982). Hypodontia in twins. *Swedish Dental Journal*.

[19] Kindelan JD, Rysiecki G, Childs WP (1998). Hypodontia: genotype or environment? A case report of monozygotic twins. *British Journal of Orthodontics*.

[20] Kantaputra P, Kaewgahya M, Kantaputra W (2014). WNT10A mutations also associated with agenesis of the maxillary permanent canines, a separate entity. *American Journal of Medical Genetics Part A*.

[21] Fukuta Y, Totsuka M, Takeda Y, Yamamoto H (2004). Congenital absence of the permanent canines: a clinico-statistical study. *Journal of Oral Science*.

[22] Harris EF, Clark LL (2008). Hypodontia: an epidemiologic study of American black and white people. *American Journal of Orthodontics and Dentofacial Orthopedics*.

[23] Davis PJ (1987). Hypodontia and hyperdontia of permanent teeth in Hong Kong school children. *Community Dentistry and Oral Epidemiology*.

[24] Fekonja A (2005). Hypodontia in orthodontically treated children. *European Journal of Orthodontics*.

[25] Sisman Y, Uysal T, Gelgor IE (2007). Hypodontia. Does the prevalence and distribution pattern differ in orthodontic patients?. *European Journal of Dentistry*.

[26] Ericson S, Kurol J (1986). Longitudinal study and analysis of clinical supervision of maxillary canine eruption. *Community Dentistry and Oral Epidemiology*.

[27] Shapira Y, Kuftinec MM (2001). Maxillary tooth transpositions: characteristic features and accompanying dental anomalies. *The American Journal of Orthodontics and Dentofacial Orthopedics*.

[28] Aydin U, Yilmaz HH (2003). Transmigration of impacted canines. *Dentomaxillofac Radiol*.

